# The influence of teacher-student proximity, teacher feedback, and near-seated peer groups on classroom engagement: An agent-based modeling approach

**DOI:** 10.1371/journal.pone.0244935

**Published:** 2021-01-07

**Authors:** Zhe Dong, Haiyan Liu, Xinqi Zheng

**Affiliations:** 1 School of Economics and Management, University of Geosciences (Beijing), Beijing, China; 2 School of Information Engineering, University of Geosciences (Beijing), Beijing, China; Universitá degli Studi di Bergamo, ITALY

## Abstract

Fostering students’ classroom engagement is a research hotspot in classroom teaching management. Enhancing classroom engagement requires consideration of the interactive effects of physical and interpersonal environments. Considering the characteristics of physical space, the teacher gives feedback on student engagement in terms of different seating positions. Further, near-seated peer group engagement has an impact, though previous research has found this to be inconsistent. The teacher and near-seated peer groups have different paths of influence on classroom engagement, and there is interplay between them. However, based on realistic classroom scenarios, it is difficult for traditional research methods to reveal how spatially heterogeneous and non-linear micro-interactions among teachers, students, and near-seated peer groups evolve into dynamic changes in macro-classroom engagement. Hence, this study utilized agent-based simulation to explore the non-linear dynamic mechanism underlying how teacher-student proximity, teacher feedback, and near-seated peer groups affect classroom engagement, thereby shedding light on the evolutionary features of classroom engagement. According to the results, the teacher’s positive feedback promoted an S-shaped increase in classroom engagement, and the closer a student sat to the teacher, the greater the increase was. The level and homogeneity of near-seated peer group engagement were predictors of changes in classroom engagement. Moreover, the proximity of students to the teacher, teacher feedback, and near-seated peer groups had a joint effect on student engagement. The compensation effect of the teacher’s positive feedback on the impact of low-engagement, near-seated peer groups was weaker than that of highly engaged, near-seated peer groups on the effects of the teacher’s negative feedback. This suggests that the model of teacher-student proximity and teacher feedback effects differed from that of near-seated peer group influence, and the two interacted and showed asymmetry.

## Introduction

The classroom is an important place for teachers to carry out teaching activities and for students to learn. Classroom teaching management is a comprehensive skill in teachers’ classroom teaching practice, whereby stimulating students’ classroom engagement is a crucial way to enhance classroom teaching effects. Classroom engagement refers to the extent that students focus on learning tasks and activities in class. The degree of students’ classroom engagement directly reflects their classroom learning motivation and affects their academic performance and social development. Hence, how to inspire and promote students’ engagement in classroom teaching has become a key issue in classroom teaching management research in recent years [1–5].

The factors that impact students’ classroom engagement are very complex. Classroom physical environmental factors such as light, acoustics, color, temperature, and seating arrangement [6] influence students’ classroom engagement. Teacher-student interactions (teacher-student, student-student) [7], teacher feedback given to students [8, 9], and peer groups [10–12] also can affect students’ classroom engagement. Different scholars have conducted useful studies on these intricate, interactive effects from various angles.

Investigations into the impact of seating arrangement on learning and classroom behavior date back to the 1920s [13]. Seating position characterizes the proximity of the teacher to the students and the proximity of students to their peers [14]. Spatial proximity may affect the interpersonal relationship between the teacher and the students, as well as the latter’s classroom engagement. Many researchers have found that the student’s proximity to the teacher is associated with classroom engagement; for example, students who sit in the front and middle of the classroom participate more and perform better than those at the back and on the sides [15–20]. The closer a student is to the teacher, the more interactions between the student and the teacher, and the stronger the student’s participation, concentration, and interest in learning. In teacher-student interactions, teacher support and positive feedback are vital [21–23]. Teachers provide more support to engaged students and less support to disengaged ones [9]. With regard to the proximity of students to their peers, research has recently begun to focus on the influence of near-seated peers on students’ academic performance. The results of these studies are inconsistent. The impact of a near-seated peer group on students’ academic performance may be positive, negative, or non-significant [24–26]. This may be because, compared to teachers, fostering student motivation is not the goal of peer groups. A near-seated peer group influences classroom engagement through a peer contagion mechanism, which is a process of convergence and amplification [27, 28]. However, the effects of peer influence vary in degree, due to different levels of student engagement and sensitivity to peer contagion. Therefore, under the effects of spatial proximity, the mechanism underlying the teacher’s impact on student classroom engagement in distinct seating positions differs from that underlying the influence of a near-seated peer group. In addition, in the classroom, teachers interact with students in different seating positions, and students in different positions interact with different, near-seated peers. There might be interaction effects between teacher-student and student-student interactions, which would complicate the evolutionary mechanism of classroom engagement.

Considering teacher-student spatial proximity, teacher feedback effects and near-seated peer group influence, the evolution of classroom engagement is a process that includes interactions among multiple individuals and multi-level feedback; it is characterized by nonlinearity and dynamics. As a result, traditional research methods have difficulty studying the evolution of classroom engagement. However, agent-based modeling (ABM) is suitable for simulating the dynamic interaction of heterogeneous individuals in complex environments, and for exploring how complex systems change over time [29]. ABM can help integrate the findings of micro-mechanisms and macro-effects in educational research [30]. It is thought that the combination of ABM with quantitative and qualitative research has the potential to reveal the dynamics of complex educational systems across a range of levels and time scales [31]. Thus, this study used the outcomes of empirical observations of student interactions with peers and teachers as well as of student behavioral engagement [7] for simulation, input, and output validation. We examined such development from interactions among the teacher, students, and peer groups in order to understand students’ classroom engagement, as well as to obtain fresh findings and provide novel hypotheses.

In sum, this study used ABM to scrutinize the impact of teacher-student proximity, teacher feedback, and near-seated peer groups on classroom engagement in order to grasp the patterns of its development. We hope these findings can help teachers to utilize seating arrangements, and to comprehend the effects of teacher feedback and peer groups. We expect to provide suggestions for maintaining students’ enthusiasm and initiative in classroom learning to enhance classroom engagement. To achieve this goal, this study first constructed an agent-based classroom engagement model, which looked at the evolution of classroom engagement under the influence of spatial proximity, teacher feedback, and peer group effects, and then validated the model. Then, through sensitivity analysis, we analyzed the impact on classroom engagement of: (a) proximity to the teacher and the teacher’s positive feedback probability, and (b) the influence of near-seated peer group engagement, and (c) the interaction mechanism between (a) and (b). Finally, the article discussed the characteristics of classroom engagement evolution, limitations, and future research suggestions, and summarized the conclusions.

## Methods

### Overview of the agent-based classroom engagement model

We constructed an agent-based classroom engagement model with NetLogo [32] to determine the influence mechanism of spatial proximity, teacher feedback, and near-seated peers on classroom engagement in classroom interactions. The model represents a classroom in which a teacher and a defined number of students engage in teaching and learning activities. Seating arrangements are shown in rows and columns. Students in the front row are closest to the teacher, while those in the back row are farthest away. The near-seated peer group of each student is set as a Moore neighborhood with the student as the center, surrounded by north, south, east, west, northeast, southeast, northwest, and southwest neighbors [33]. In classroom teaching and learning, students either interact with teachers, near-seated peers, or do not interact at all. Students adjust their level of classroom engagement based on teacher feedback and the social influence of near-seated peers, resulting in either engagement or disengagement. The input variables here are the proximity of the teacher and the students, teacher feedback, and near-seated peer group engagement; the output variables are the time proportions of classroom engagement and disengagement (see the section “Model inputs and outputs” for details).

As shown in Table 1, in the initialization phase, the model sets the classroom characteristics, including the physical environment (seating arrangements, class size, etc.) and the main properties of the teacher and the students (see the section “Parameter initialization” for details). The proportions of teacher-student and student-student interactions are set based on empirical outcomes [7]. The teacher agent has positive and negative feedback attributes, student agents have classroom engagement attributes, and links between agents have relatedness attributes that are highly associated with classroom engagement [34]. Under model dynamics, three changes are hypothesized in accordance with the behavioral rules (see the section “Behavioral rules” for details).

**Table 1:** Model overview.

**Initialization**		
(1) Number of agents and seating arrangements	(2) Characters of agents	
	Probability of teacher’s positive feedback	Probabilities of student engagement/disengagement
		Engagement composition
(3) Relationships among agents	a. Teacher-student	b. Student-peer group
	Physical distance	Near-seated peer group
	Relatedness	Relatedness
	Interaction probability	Interaction probability
**Behavioral rules**		
(1) Seating proximity and teacher effect	(2) Near-seated peer group influence	(3) Engage/Disengage alone
**Model outcomes**		
(1) Classroom engagement and disengagement time	(2) Engagement time rate	(3) Engagement time proportion distribution

Hypothesis 1: Classroom engagement is affected by the proximity of the teacher and the students, as well as teacher feedback. The closer the distance between the teacher and the students and the more positive the teacher’s feedback, the higher the classroom engagement.
Hypothesis 2: Classroom engagement is affected by the influence of near-seated peer groups. Near-seated peer group engagement (disengagement) contributes to higher (lower) classroom engagement.
Hypothesis 3: Spatial proximity, teacher feedback, and peer groups jointly predict classroom engagement.

We propose that students who both receive the teacher’s positive feedback (TPF) and have an engaged near-seated peer group have the highest levels of classroom engagement; the lowest levels of classroom engagement result from the teacher’s negative feedback (TNF) and near-seated peer group disengagement, showing cumulative effects. We expect that TPF buffers the negative impact of a disengaged, near-seated peer group on classroom engagement, and students closer to the teacher receive a stronger buffering effect; near-seated peer group engagement weakens the negative impact of TNF on classroom engagement, and peer groups with more engagement show a stronger weakening effect, indicating interactive effects.

In order to investigate the iterative effect and continuous changes in classroom engagement, the study carried out 2,000 model steps. Each step represents a five-minute segment according to the behavioral engagement observation outcomes of [7]. For changes in student engagement from moment to moment, the researcher logged the student’s activities every 5 min on a log, which asked for several pieces of information, such as what the student was doing, with whom the student was interacting, and whether the student was on task or off task. After 2,000 steps, the changes in classroom engagement tended to stabilize. At each step, the model reported the cumulative frequency and proportions of student engagement and disengagement at the individual student level, as well as the mean and standard deviation of the rate of classroom engagement at the regional and class levels. Subsequently, we validated and calibrated the model by comparing it with the behavioral engagement results of [7]. Further, we conducted a sensitivity analysis, which specifically examined changes in classroom engagement when modifying class size, the distance between the teacher and the students, TPF probability, student engagement/disengagement probability and composition, and sensitivity to the positive and negative influences of the teacher and near-seated peers.

### Parameter initialization

With reference to previous studies on the impact of seating position on classroom engagement [35], classroom seating was arranged randomly in order to control for the self-selection effect. Each student had their own location coordinates. The first row was closest to the teacher, and the last row was the farthest away. Student location was also used to identify near-seated peers. In order to obtain a sufficient group of peers, we selected a class size of 49 students and arranged seats on a 7 x 7 grid.

Each student had the attributes of engagement and disengagement—the probability of engagement/disengagement, ranging from 0% to 100%. In the random model, the student’s initial probability of engagement was 50% (i.e., an equal probability of exhibiting engagement or disengagement), and the student’s previous engagement status was assumed to be unknown. In the model that controlled for the proportions of high- and low-engaged students, high-engaged students’ initial engagement probability was set to 75%, while that of low-engaged students was set to 25%. The teacher had the attributes of giving positive and negative feedback. When faced with student engagement and disengagement behavior, the teacher gave positive or negative feedback. TPF probability—the proportion of positive feedback in the sum of positive and negative feedback—was set with a value range of 0–1.

There were relatedness attributes between the teacher and each student, as well as between each student and each of the eight members of the near-seated peer group. The initial setting was 0, and the value range was -10–10. The positive values reflected warmth, friendliness, and acceptance, while the negative values reflected hostility, dislike, and rejection. The larger the value, the stronger the relatedness. The initial value was calculated based on the results of observations of student interactions in [7]. The probabilities of student-teacher interaction *P*_*t*−*s*_, peer interaction *P*_*s*−*s*_, and no interaction *P*_*alone*_ were 18.7%, 27.9%, and 53.4%, respectively, and the sum was 100%.

### Behavioral rules

Classroom engagement changes during interactions between the teacher and the students, and between students and their peers. The proximity of students to the teacher and teacher feedback both play a role in teacher-student interactions, while near-seated peer groups influence classroom engagement through student-student interactions. According to the initial set of teacher-student interaction, student-student interaction, and non-interaction probabilities, within five minutes of each time step, the teacher interacts with any number of students, any number of other students interacts with the neighboring companion group, while the remaining students do not interact with anyone. During this period, some near-seated peer group members who had affected the central student may have interacted with the teacher. The rules for interactions between the teacher and the students, and between students and their peers, are described below.

#### Rules for the impact of teacher-student proximity and teacher feedback

Teacher-student proximity and teacher feedback affect classroom engagement in teacher-student interaction. The row number of student seats represented the distance to the teacher. The distance *d* from the first row to the teacher was 1, and the distance *d* from the seventh row to the teacher was 7. The distance between the students and the teacher negatively predicts teacher-student interactions, such as verbal participation and nonverbal eye contact [35]. The probability of the teacher interacting with each row of students was calculated as follows:

$$p_{t-s}=P_{t-s}+\alpha (d-\sum_{i=1}^{r}i/r)$$

where *P*_*t-s*_ refers to the average probability of teacher-student interactions, *r* refers to the total number of seated rows, and *α* is the predictive coefficient of teacher-student distance to teacher-student interaction, with a value of -1–0.

The teacher interacted with any student in each row. Students in the interaction showed engagement or disengagement, and the teacher gave positive or negative feedback. Classroom engagement affects teacher support and feedback [8, 9]. Student *i*’s engagement makes the teacher’s feedback more positive, thereby increasing the strength of teacher-student relatedness; student disengagement triggers more negative feedback from the teacher, thereby reducing the strength of teacher-student relatedness. The perceived strength of relatedness positively predicts classroom engagement, directly altering the probability of student *i*’s engagement and disengagement. People tend to pay attention to negative events, negative interactions, or the negative personal qualities of others [36]. We assumed that positive and negative teacher-student relatedness had an asymmetric effect on classroom engagement. Here, we used positive and negative teacher-student relatedness thresholds to control for the asymmetric effect. The positive relatedness threshold was supposed to be higher than the negative one. The probability and duration of student engagement and disengagement changed between *t* and *t+*1 time according to the following rules:

a. Rules for changes in the teacher-student relatedness strength *rs*_*t-si*_ and the duration of engagement and disengagement:
  If student *i* engages with a *enprob*_*i*_, then *engage_time*_*i*_ = *engage_time*_*i*_ + 1.
     If the teacher gives positive feedback, then *rs*_*t*−*si*_ = *rs*_*t*−*si*_ + *m*.
     If the teacher gives negative feedback, then *rs*_*t*−*si*_ = *rs*_*t*−*si*_ − *n*.
  If student *i* disengages with a *disenprob*_*i*_, then *disengage_time*_*i*_ = *disengage_time*_*i*_ + 1.
     If the teacher gives positive feedback, then *rs*_*t*−*si*_ = *rs*_*t*−*si*_ + *n*.
     If the teacher gives negative feedback, then *rs*_*t*−*si*_ = *rs*_*t*−*si*_ − *m*.
b. Rules for changes in perceived teacher-student relatedness *pr*_*t-si*_:
  If *rs*_t−si_ ≥ 0, then ${pr}_{t-si}=10(1-e^{\beta *{rs}_{t-si}})$, *β∈*(−0.1, 0).
    If *rs*_t−si_ < 0, then ${pr}_{t-si}=-10(1-e^{\beta *{rs}_{t-si}})$, *β∈*(0, 0.1).
c. Rules for changes in engagement *enprob*_*i*_ and disengagement probabilities *disenprob*_*i*_:
  If *pr*_*t−si*_ > *pr*_*ts_threshold*_ *or pr*_*t−si*_ < *nr*_*ts*___*threshold*_:
     then *enprob*_*i*_ = *initial_enprob*_*i*_ + *γ* * *pr*_*t−si*_, *γϵ*(0,1),
     and *disenprob*_*i*_ = 1 − *enprob*_*i*_.

where *m* and *n* refer to the change in relatedness strength and *m* > *n*. *pr*_*ts_threshold*_ and *nr*_*ts_threshold*_ refer to the positive and negative teacher-student relatedness thresholds, respectively; *pr*_*ts_threshold*_ > 0, *nr*_*ts*___*threshold*_ ≤ 0 and *pr*_*ts_threshold*_ > |*nr*_*ts*___*threshold*_|. *β*, *γ* are adjustment factors and adjust the change rate of perceived teacher-student relatedness and classroom engagement probability. *initial_enprob*_*i*_ refers to the initial value of student *i*’s engagement probability.

#### Rules for near-seated peer group influence

Students were exposed to peer group influence while interacting with their near-seated peers. The similarity between students and their peers affects peer relationships. If the student and the near-seated peer perform more similarly in class, their relatedness will be stronger and more positive. In the opposite case, peer relatedness will decrease. The model identified different types of peer groups—friendly or hostile—through the attributes and strengths of student *i*’s relatedness with the near-seated peer group. Near-seated classmates could have an initial negative impact on student classroom engagement before becoming friends [25]. We supposed that students and friendly, near-seated peer groups had the same levels of classroom engagement. The overall engagement of the near-seated peer group positively predicted the probability of the student’s classroom engagement. We also assumed that the performance of students was contrary to the general engagement of a hostile, near-seated peer group. Under hostile conditions, students disengaged more when the near-seated peer group was more engaged. Since individuals are more sensitive to negative influences, higher positive and lower negative peer relatedness thresholds were used to control for the impact of friendly and hostile neighboring peer groups on classroom engagement. The probability and duration of student engagement and disengagement changed between *t* and *t*+1 time in accordance with the following rules:

a. Rules for changes in student *i*’s relatedness strength with peer *j rs*_*si-pj*_ and the duration of engagement and disengagement:
  If student *i* engages with a *enprob*_*j*_, then *engage_time*_*i*_ = *engage_time*_*i*_ + 1.
     If peer *j* engages with a *enprob*_*j*_, then *rs*_*si*−*pj*_ = *rs*_*si*−*pj*_ + *m*.
     If peer *j* disengages with a *disenprob*_*j*_, then *rs*_*si*−*pj*_ = *rs*_*si*−*pj*_ − *m*.
  If student *i* disengages with a *disenprob*_*i*_, then *disengage_time*_*i*_ = *disengage_time*_*i*_ + 1.
     If peer *j* engages with a *enprob*_*j*_, then *rs*_*si*−*pj*_ = *rs*_*si*−*pj*_ − *m*.
     If peer *j* disengages with a *disenprob*_*j*_, then *rs*_*si*−*pj*_ = *rs*_*si*−*pj*_ + *m*.
b. Rules for changes in perceived peer relatedness *pr*_*si-pj*_:
  If *rs*_si−pj_ ≥ 0, then ${pr}_{si-pj}=10(1-e^{\beta *{rs}_{si-pj}})$, *β∈*(−0.1, 0).
    If *rs*_si−pj_ < 0, then ${pr}_{si-pj}=-10(1-e^{\beta *{rs}_{si-pj}})$, *β∈*(0, 0.1).
c. Rules for changes in engagement *enprob*_*i*_ and disengagement probabilities *disenprob*_*i*_:
  If median (*pr*_*pg*_) > *pr*_*peer_threshold*_:
     If Σ*engage_time*_*i*_ > Σ*disengage_time*_*j*_:
      then *enprob*_*i*_ = *initial_emprob*_*i*_ + *γ* * medium (*pr*_*pg*_), *γ∈*(0,1).
     Or else:
      then *disenprob*_*i*_ = *initial_disemprob*_*i*_ + *γ* * medium (*pr*_*pg*_), *γ∈*(0,1).
  If $median({pr}_{pg})<{nr}_{peer_{threshold}}$:
     If Σ*engage_time*_*j*_ > Σ*disengage_time*_*j*_:
      then *disenprob*_*i*_ = *initial_disemprob*_*i*_ + *γ* * medium (*pr*_*pg*_), *γ∈*(0,1).
     Or else:
      then *enprob*_*i*_ = *initial_emprob*_*i*_ + *γ* * medium (*pr*_*pg*_), *γ∈*(0,1)

where *pr*_*pg*_ refers to all perceived relatedness of student *i* with the near-seated peer group members. *pr*_*peer_threshold*_and *nr*_*peer_threshold*_ refer to positive and negative peer relatedness thresholds, respectively. *pr*_*peer_threshold*_ > 0, *nr*_*peer_threshold*_ ≤ 0 and *pr*_*peer_threshold*_ > |*nr*_*peer_threshold*_|. *m*, *n*, *β*, *γ* and *intial_enprob*_*i*_ are the same as above.

#### Rules for no interactions

When students did not interact with other agents, the engagement probability of students at time step *t* was the mean value of the engagement probability under the influence of the teacher and peer groups at time *t*-1. From *t* to *t*+1, the probability and duration of student *i*’s engagement and disengagement changed according to the following rules:

If random 100 > *enprob*_*i*_:
   Student *i* engages and then *engage_time*_*i*_ = *engage_time*_*i*_ + 1.
Or else:
   Student *i* disengages and then *engage_time*_*i*_ = *engage_time*_*i*_ + 1.

### Model inputs and outputs

In order to examine the influence of teacher-student proximity, teacher feedback, and near-seated peer groups on classroom engagement, the input of the model included the following:

(a) The students’ proximity to the teacher. Here, the front and back rows represent the distance between the teacher and the students. The front rows included the first four rows, and the back rows included the last three rows.
(b) The probability of TPF, which controlled whether the teacher gave more positive or negative feedback on classroom engagement. A greater probability meant more positive feedback.
(c) The degree of engagement of the near-seated peer group, which included the characteristics of engagement probability and homogeneity. Since every student in the class has a near-seated peer group, the average and difference of class-level engagement probability were used to control for the engagement of the near-seated peer group, resulting in the following four scenarios: (1) homogeneous low engagement (Homo_Low): the entire class is comprised of low-engagement students, with an initial engagement probability of 25%; (2) homogeneous medium engagement (Homo_Medium): the entire class is comprised of medium-engagement students, with an initial engagement probability of 50%; (3) homogeneous high engagement (Homo_High): the entire class is comprised of high-engagement students, with an initial engagement probability of 75%; (4) heterogeneous in engagement (Hetero): the number of low- and high-engagement students in the class is equal. The average level of initial classroom engagement in the heterogeneous and homogeneous medium classes is the same.

The model included three outputs:

(a) The duration of classroom engagement, disengagement time, and the engagement rate of student *i*. The classroom engagement rate represented the level of student engagement. The formula is as follows:
  engageratei=engage_timei(engage_timei+disengage_timei)
(b) Classroom engagement rate in the front and back rows, of the class overall, and in interactions with the teacher, peers, and non-interactions. The formula is as follows:
  Engagementrate=∑engage_timei(∑engage_timei+∑disengage_timei)
  $$Engag{ement}_{rate}+Disengag{ement}_{rate}=100\%$$
(c) Distribution of classroom engagement, described by student number distribution according to engagement rate: high engagement (75% or greater), high-medium engagement (50% or greater and less than 75%), low-medium engagement (25% or greater and less than 50%), and low engagement (less than 25%).

### Model validation

**Fig 1:**
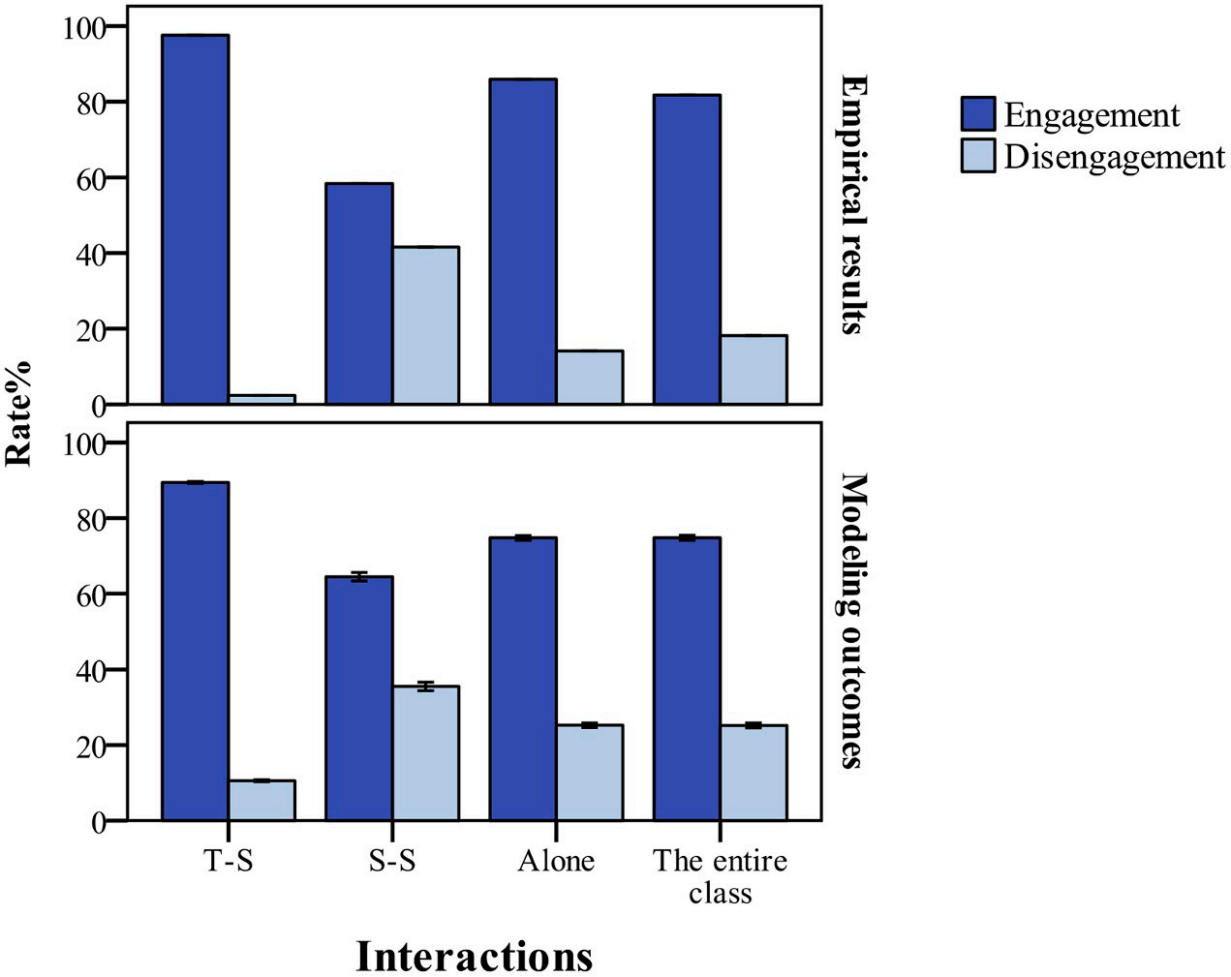
Empirical results vs. modeling outcomes on classroom engagement in student interaction scenarios. The trends of engagement vs. disengagement rates within and between three types of student interactions are similar. “T-S” refers to student interactions with the teacher, “S-S” refers to student interactions with peers, and “Alone” refers to no interactions. Error bars in all simulation figures indicate one standard error (i.e., the sample standard deviation divided by the square root of the number of replications).

**Fig 2:**
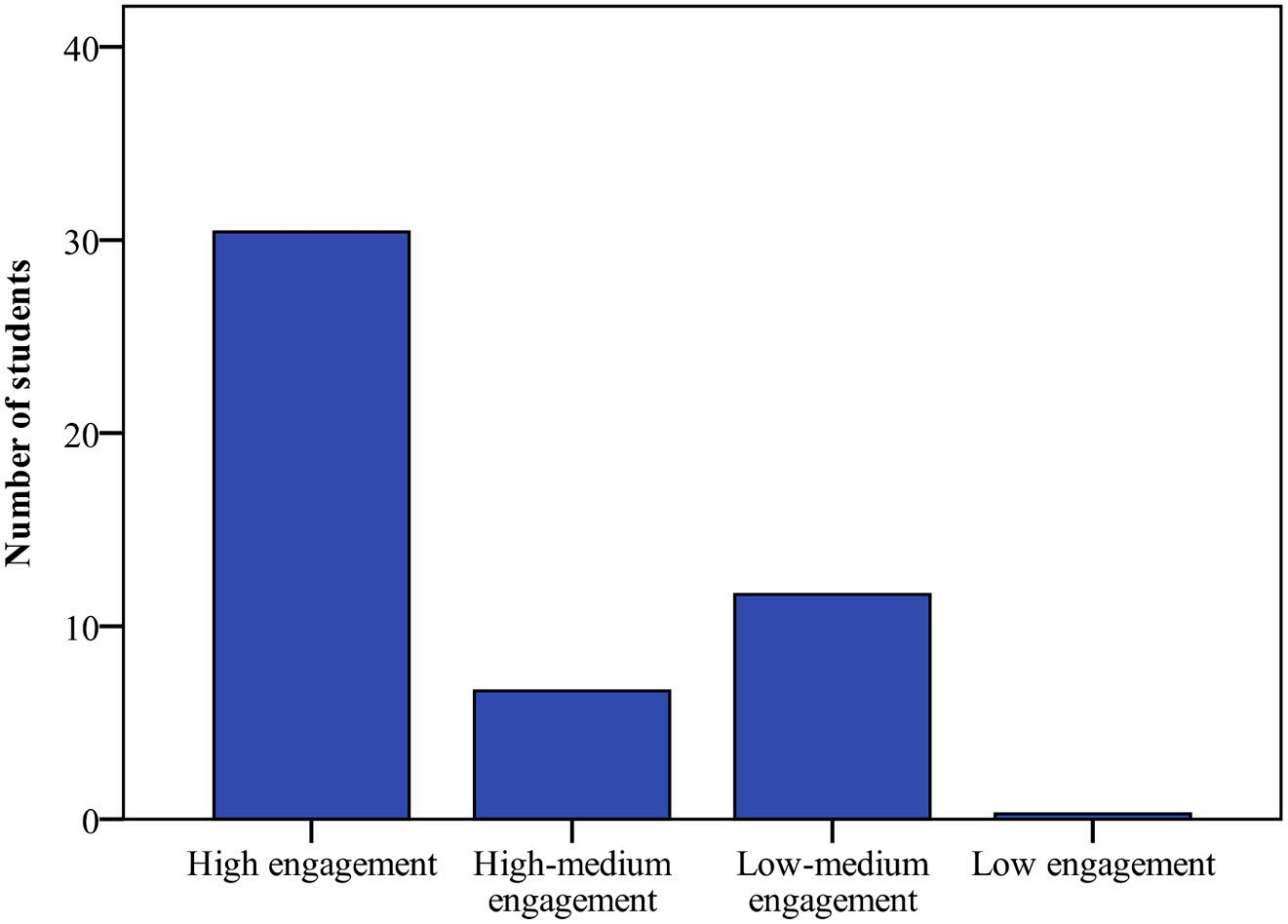
Simulated distribution of classroom engagement in model validation. Students’ classroom engagement rates include four levels: high, high-medium, low-medium and low engagement. The number of students for each level is depicted.

Validation is the process of determining whether there is correspondence between the implemented model and reality. This study validated the results of the model by comparing them to Nguyen et al.’s [7] behavioral engagement study, in which the observed results describe the proportions of active/passive engagement and disengagement when students interacted solely with the teacher or other students, interacted with both the teacher and their peers, and did not interact with anyone. The present study did not focus on simultaneous interactions with both students and the teacher, so the simulation outcomes compare the proportions of engagement (active plus passive engagement) and non-engagement in three scenarios: interactions with the teacher, interactions with peers, and no interactions. The model parameters were set, as shown in Table 2. The model uses behavioral space for simulation experiments. As outlined in Fig 1, the trends of the empirical and simulation results were similar. When interacting with the teacher, the engagement percentage was higher than when interacting with students. Hence, the parameter settings demonstrate that the teacher generally gave more positive feedback in interactions, and students had a moderate relatedness threshold for teacher-student and peer influence. Although classroom engagement time could reach about 75% for the entire time, there were still a few disengagement behaviors that deserve the teacher’s attention. In a class of 49 students, there were about 12 students (Low-medium engagement: 11.65±2.14; Low engagement: 0.28±0.55) whose engagement time was less than 50% (Fig 2).

**Table 2:** Parameters of the simulation.

Parameters	Model validation	Sensitivity analysis	
		Hypothesis testing	Other robustness analysis
Teacher-student interaction probability	18.7%	18.7%	18.7%
Peer interaction probability	27.9%	27.9%	27.9%
Non-interaction probability	53.4%	53.4%	53.4%
Teacher-student and peer relatedness thresholds	Positive: 5	Positive: 5	Positive: 1, 3, 5, 7, 9
	Negative: 0	Negative: 0	Negative: 0
Teacher’s positive feedback probability	0.6	0.2, 0.4, 0.6, 0.8	0.6
Classroom engagement composition	All 50%	All 25%, all 50%, all 75%	All 50%
		half 25% and half 75%	
Class size	49	49	25, 49, 81
Seating arrangement (row * column)	7*7	7*7	5*5, 7*7, 9*9
Number of replications	100	30	30
Length of run	2000 timesteps	2000 timesteps	2000 timesteps

## Results

To examine model hypotheses, sensitivity analysis separately explored (1) the influence of the proximity of students to the teacher and teacher feedback on classroom engagement, (2) the influence of near-seated peer groups on classroom engagement, and (3) the joint effect of teacher-student proximity, teacher feedback, and near-seated peer groups. The sensitivity analysis parameter settings are displayed in Table 2, and the sensitivity analysis results of other parameters are portrayed in the S2 Appendix.

### Influence of teacher-student proximity and teacher feedback on classroom engagement

We controlled for TPF probability in teacher-student interactions. The teacher in the model gives positive or negative feedback based on this probability. The descriptive statistics of classroom engagement for teacher-student proximity and teacher feedback effects are depicted in Table 3. The classroom engagement rate of the entire class increased with a rise in TPF. TPF probability differed in the change in classroom engagement of the entire class (F_3, 116_ = 2426.551, p < .001, η^2^ = .984). The results of the LSD test indicated significant differences between each level.

**Table 3:** Descriptive statistics of classroom engagement for teacher-student proximity and teacher feedback effects.

	Teacher’s positive feedback probability			
	0.2	0.4	0.6	0.8
Front rows	14.66 ± 2.33	19.50 ± 3.41	79.59 ± 5.12	89.28 ± 4.08
Back rows	22.55 ± 4.13	28.00 ± 4.03	69.75 ± 5.92	76.47 ± 5.89
The entire class	19.17 ± 2.75	24.36 ± 3.19	73.97 ± 4.25	81.96 ± 4.12

The results of the paired t-test revealed that the difference in classroom engagement between the front and back rows was significant at all levels of TPF probability (p < .001). When the probability was less than 50%, classroom engagement in the front rows was lower than in the back rows (20%: t_29_ = -10.066, p < .001, two-tailed, d = -2.01; 40%: t_29_ = -11.439, p < .001, two-tailed, d = -1.85). When the probability was greater than 50%, classroom engagement in the front rows was higher than for the back rows (60%: t_29_ = 7.340, p < .001, two-tailed, d = 2.00; 80%: Wilcoxon’s sign rank test Z = -4.741, p < .001).

### Influence of near-seated peer groups on classroom engagement

We controlled for the engagement of near-seated peer groups through class engagement composition (the average level and differences). The study compared and examined the changes in classroom engagement in low, medium, and high engagement homogeneous and heterogeneous engagement groups. The Kruskal-Wallis test results demonstrated that the classroom engagement rates of the entire class differed significantly according to the composition of class engagement probability (χ^2^ = 111.57, p < .001). The classroom engagement rate in descending order is as follows: high-engagement homogeneous group (97.06 ± 1.42), medium-engagement homogeneous group (75.17 ± 4.21), heterogeneous engagement group (57.96 ± 1.25), and low-engagement homogeneous group (21.80 ± 0.77). Near-seated peer groups had the highest level of engagement and engagement similarity in the high-engagement homogeneous class, whereby the level of classroom engagement finally became the highest. Nevertheless, near-seated peer groups had the lowest level of engagement and the same homogeneity in the low-engagement homogeneous class, whereby the students had the lowest classroom engagement. This suggests that the average level of near-seated peer group engagement made a greater contribution to changes in the level of classroom engagement.

**Fig 3:**
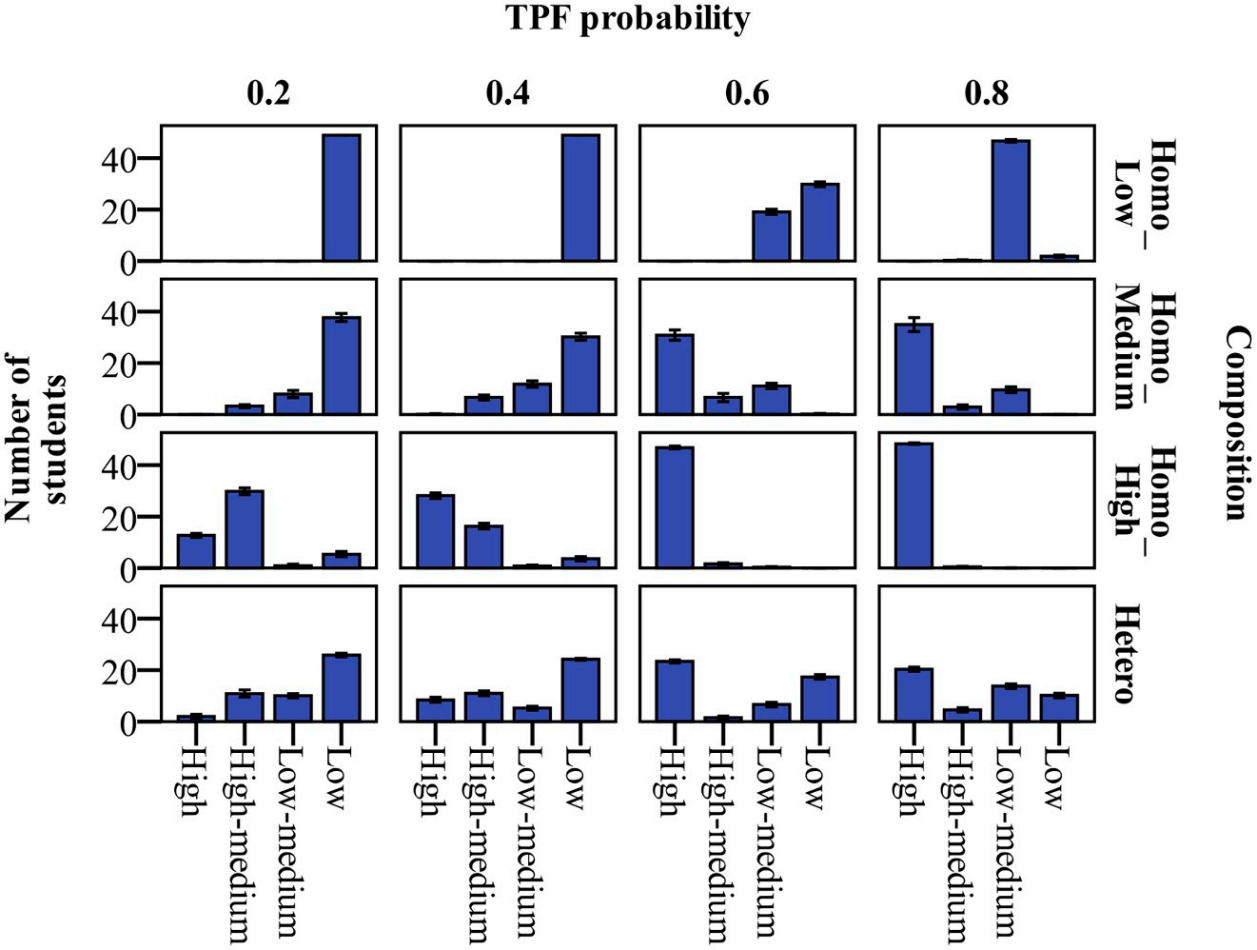
Simulated distribution of classroom engagement under teacher feedback effects and near-seated peer group influence. “TPF” = teacher’s positive feedback. “Composition” refers to class engagement composition, including four scenarios: low-engagement homogeneous (Homo_Low), medium-engagement homogeneous (Homo_Medium), and high-engagement homogeneous (Homo_High) and heterogeneous groups (Hetero: half low- and half high-engagement students). Under the effects of teacher’s positive feedback probability and class engagement composition, the numbers of students with high, high-medium, low-medium and low classroom engagement rate are presented.

The study compared the distribution of the classroom engagement rate. The results are described in the column of TPF probability = 0.6, as outlined in Fig 3. In the homogeneous group, student engagement changed from distributed in the low-medium- and low-engagement groups to clustered in the high-engagement area as the initial level of classroom engagement increased. Although the initial level of classroom engagement was the same in the heterogeneous group and the medium-engagement homogeneous group, the students in the heterogeneous group were clustered at both high and low engagement levels, and the numbers were the same (high engagement level: 23.4 ± 1.67 people; low engagement level: 17.4 ± 2.46 people). The homogeneity and heterogeneity of near-seated peer group engagement affected the concentration and difference trends of the classroom engagement rate.

The joint effects of teacher-student proximity, teacher feedback and near-seated peer group

#### Cumulative effects

**Fig 4:**
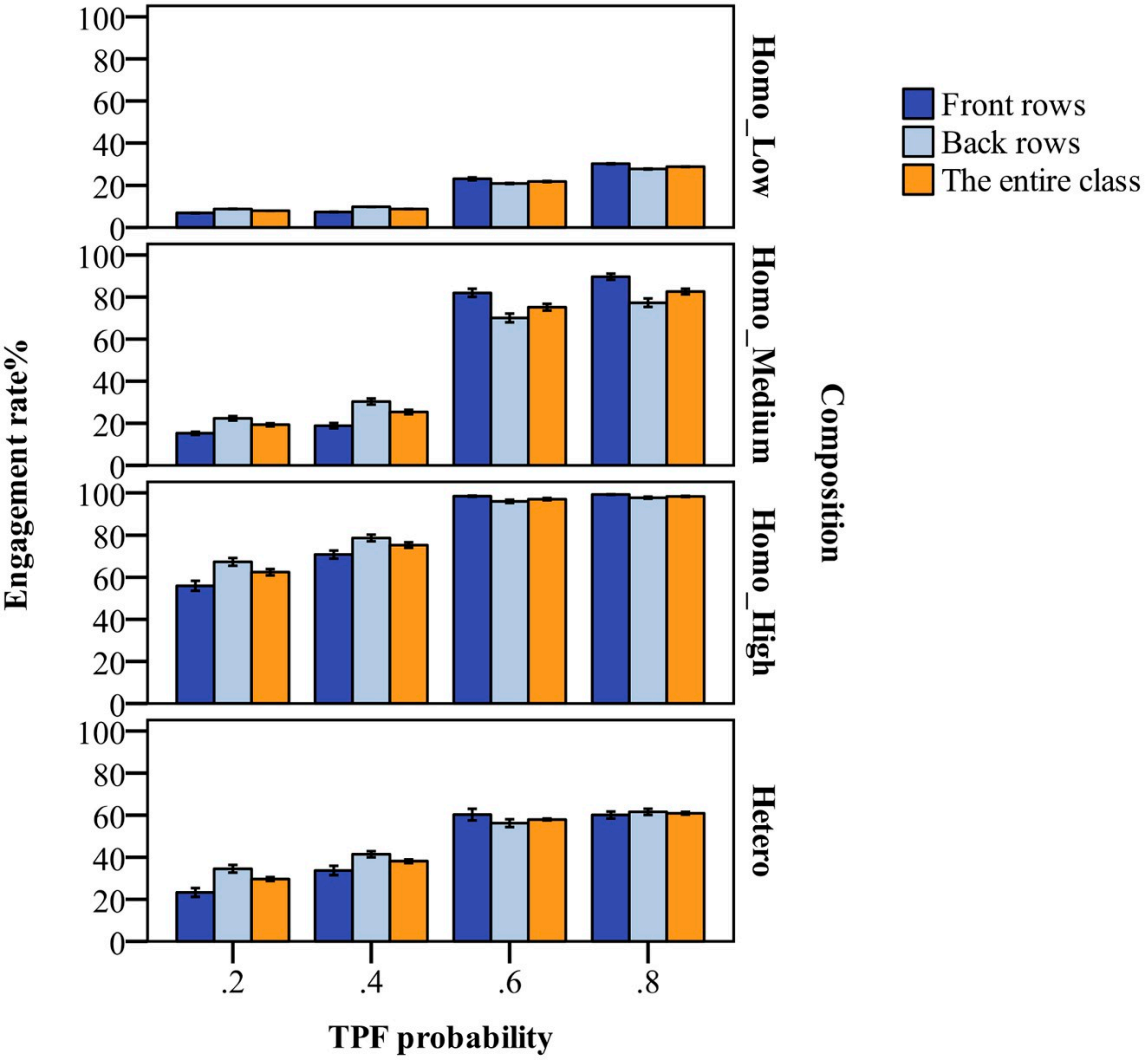
Simulated classroom engagement of front and back rows and the entire class under teacher feedback effects and near-seated peer group influence. The meanings of TPF, Composition, Homo_Low, Homo_Medium, Homo_High and Hetero are the same to those in Fig 3. The classroom engagement rates of front vs. back rows and the entire class are jointly impacted by the teacher’s positive feedback probability and classroom engagement composition.

The cumulative effect test was carried out in two steps. First, we examined the influence of teacher feedback and near-seated peer groups on classroom engagement (Figs 3 and 4). The results indicated that: (1) Teacher feedback had an obvious, consistent influence on classroom engagement. Classroom engagement increased with a rise in TPF. The change was manifested as an S-shaped curve growth process, which exhibited rapid growth between 0.4 and 0.6. (2) In the homogeneous engagement groups, classroom engagement and, especially, high-engagement students increased with a rise in initial classroom engagement level, while the difference in classroom engagement was apparent among students in the heterogeneous groups. (3) The highest classroom engagement was found in the high-engagement homogeneous groups, which received the most positive feedback from the teacher, while the lowest classroom engagement was found in the low-engagement homogeneous groups, when the teacher mostly gave negative feedback. Compared to the medium-engagement homogeneous groups, the heterogeneous groups showed less classroom engagement.

Subsequently, we looked at whether the distance between the teacher and the students affected the cumulative effect in the previous step, comparing the changes in the level of the classroom engagement of students in the front and back rows (Fig 4). The outcomes show that, in general, the changes in classroom engagement in the front and back rows were consistent with those in the entire class, showing an S-shaped enhancement trend. More precisely, when the teacher gave more negative feedback, the students in the front row had less classroom engagement than those in the back row. On the contrary, when TPF was higher, classroom engagement in the front rows was higher than that in the back. In addition, in the low- and high-engagement homogeneous and heterogeneous groups, when the teacher’s feedback was more positive, the difference in classroom engagement between the front and back rows was not obvious. Therefore, when the teacher’s negative feedback was dominant and the engagement level of the near-seated peer groups was low, the front-row students’ classroom engagement was relatively low; when the teacher’s feedback was more positive and the near-seated peer group’s engagement level was high, classroom engagement in both the front and back was high.

#### Interactive effects

**Fig 5:**
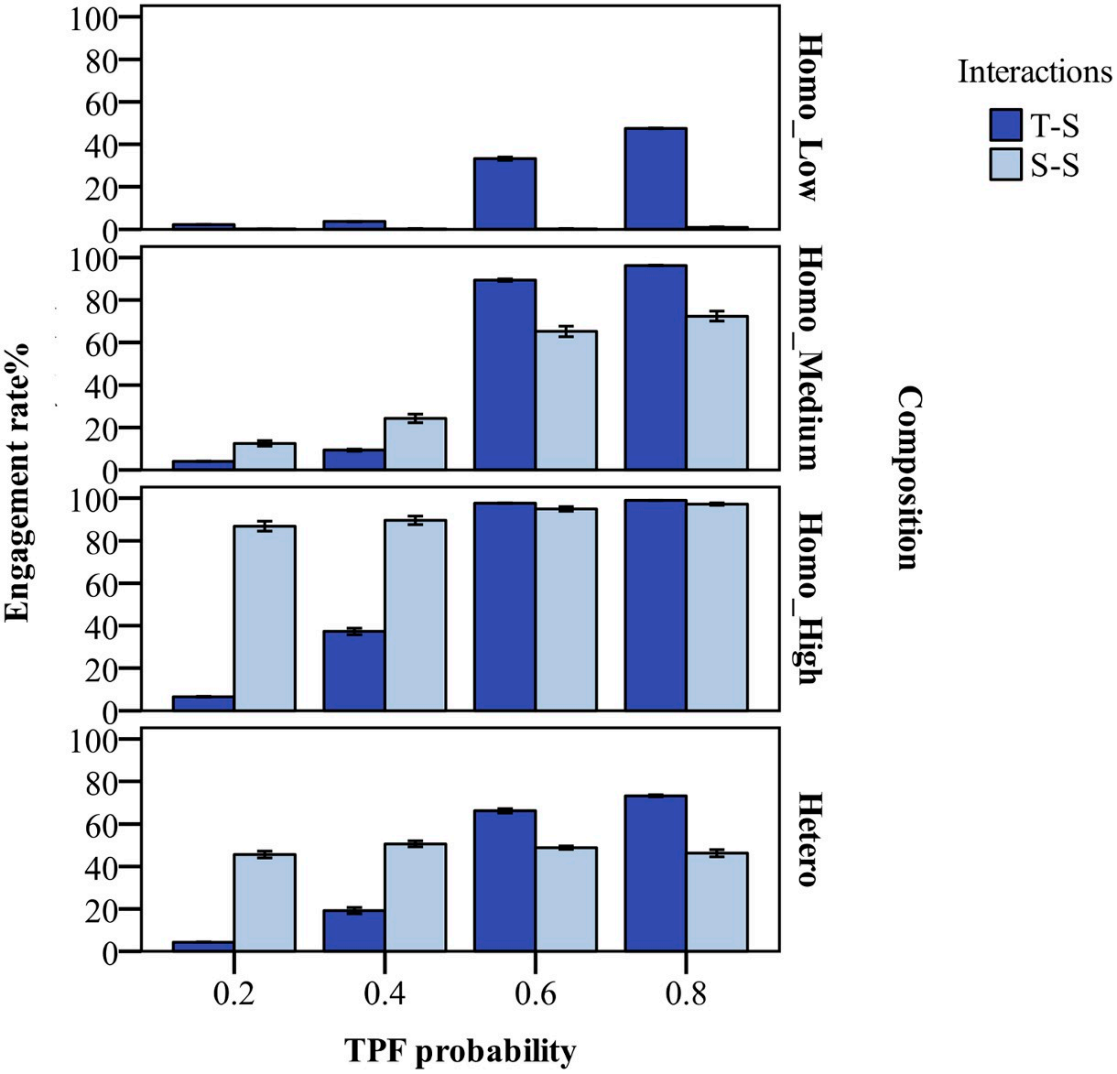
Simulated classroom engagement in interactions with the teacher and peers under teacher feedback effects and near-seated peer group influence. The meanings of TPF, Composition, Homo_Low, Homo_Medium, Homo_High and Hetero are the same to those in Fig 3. T-S and S-S refer to teacher-student and student-student interactions respectively. The effects of teacher’s positive feedback probability and class engagement composition on classroom engagement rates are asymmetrical and interactive.

In model validation, both the simulation and empirical research findings indicated that classroom engagement in student-student interactions was less than that of teacher-student interactions. In order to examine the interactive effects, the study analyzed classroom engagement development in the front and back rows, as well as of the entire class. Further, we explored classroom engagement in both teacher-student interactions and student-student interactions (Fig 5).

The results signaled that when the teacher’s feedback was predominantly negative, an increase in the engagement level of near-seated peer groups helped to improve classroom engagement. When the engagement level of near-seated peer groups was very low, an increase in the teacher’s positive feedback was helpful to boost classroom engagement, which improved the classroom engagement of front-row students even more. This reflected the mutual compensation of teacher feedback effects and near-seated peer group influence. However, compared with the compensation effect of the teacher’s positive feedback, high-engagement, near-seated peer groups were likely to enhance classroom engagement more obviously. In the low-engagement homogenous groups, an increase in the probability of the teacher’s positive feedback could increase classroom engagement to a low-medium level. When the teacher gave more negative feedback, the overall improvement of near-seated peer group engagement could lead to high-medium and even high classroom engagement. With more positive feedback from the teacher, the average level of classroom engagement in the medium-engagement, homogeneous groups exceeded that of the heterogeneous groups, while when receiving more negative feedback, the level of classroom engagement in the heterogeneous groups was higher.

Fig 5 shows the changes in classroom engagement under different interaction modes. When there was less positive feedback from the teacher in the classroom, the high engagement of near-seated peer groups not only enhanced classroom engagement in interactions with peers, but also increased the engagement rate in teacher-student interactions. Positive feedback from the teacher could improve classroom engagement in teacher-student interactions, but the enhancement in classroom engagement resulting from peer interactions was not evident, except in medium-engagement homogeneous groups.

## Discussion

This research aimed to explore the influence mechanism of spatial proximity, teacher feedback, and peer groups on changes on classroom engagement. According to the findings of previous empirical studies, we constructed an agent-based classroom engagement model, and explored the feedback mechanism of teacher feedback and peer influence on classroom engagement, incorporating the characteristics of physical space. We scrutinized the joint effects of the physical proximity of students to the teacher, teacher feedback, and near-seated peer groups on classroom engagement evolution.

### Evolution of classroom engagement under the influence of teacher-student proximity and teacher feedback

The research results partially supported Hypothesis 1. An increase in TPF probability predicted an increase in classroom engagement. The teacher’s positive feedback shaped classroom engagement by nurturing the students’ sense of relatedness [34]. Both the front and back rows and the entire class’s engagement in the classroom revealed an S-shaped curve growth trend, growing rapidly when TPF probability was about 50%. The notable finding here is that the front-back differences in student engagement each had an inflection point between 40% and 60%. Because the front-row students interacted more with the teacher, when the teacher gave less positive feedback, the teacher-student relationship involving front-row students worsened, compared to that involving back-row students. Finally, classroom disengagement in the front accumulated more than in the back. During this period, students in the front row had less and less classroom engagement, which reduced teacher support. Compared with the students in the back rows, the students in the front rows were more likely to form a vicious cycle in interactions with the teacher, accelerating the reduction of classroom engagement.

It is essential for teachers to give more positive feedback to students whether they are engaged or not. When the ratio of positive to negative feedback was around 1:1, classroom engagement increased rapidly. However, even if the ratio of the teacher’s positive to negative feedback reached 4:1, there was still a small number of students with classroom engagement below 50%, and significant differences in classroom engagement between front and back row students. When the ratio of positive to negative interactions exceeded 5:1, students’ engagement was greatly improved, and teachers could increase the proportion of positive interactions through training, such as a 5:1 proactive classroom management strategy [37].

### Evolution of classroom engagement under the influence of near-seated peer groups

We investigated the impact of near-seated peer groups on classroom engagement by controlling for the average level and differences in classroom engagement. The results verified Hypothesis 2. A high level of engagement of near-seated peer groups enhanced students’ classroom engagement, while the heterogeneity of near-seated peer group engagement led to the polarization of classroom engagement. One reason for this might be that the relationship between students and peers also became polarized, leading to hostility and division within groups. Another reason might be the students’ inconsistent classroom performance, which can contribute to the inability to form close friendships. The classroom engagement of near-seated peers who were not befriended negatively affected students’ classroom engagement [25].

Near-seated peer groups are easy to identify, and teachers can make timely, targeted interventions based on the group’s classroom engagement, such as by altering seating arrangements. Teachers could place disengaged peer groups farther apart or surround disengaged individuals with engaged groups to improve students’ classroom engagement. Further, good peer relationships can have both negative and positive effects. While encouraging students to interact with their high-engagement, near-seated peer groups, teachers should also pay attention to possible high-disengagement group aggregation in the classroom.

### Evolution of classroom engagement under joint effects of study factors

This research attempted to uncover the mechanism underlying the joint effects of teacher feedback and peer group influence on classroom engagement. The model included the feedback effect of classroom engagement on the roles of the teacher and peer groups and considered the impact of spatial proximity. The model simulations obtained outcomes similar to those of [12] and the Hypothesis 3 was verified. The effects on classroom engagement of teacher feedback and near-seated peer group influence accumulated. The most engaged students were those who received the most positive feedback from the teacher and were influenced by high-engagement, near-seated peer groups, while most classroom disengagement was found when the teacher often gave negative feedback on student engagement or disengagement, and near-seated peer groups were disengaged. In line with these interactive effects, the teacher’s positive feedback buffered the negative influence of disengagement in near-seated peer groups to a certain extent, and the influence of near-seated, high-engaged peer groups could make up for the impact of the teacher’s negative feedback on classroom engagement.

Meanwhile, the research also yielded some interesting, novel findings. Teacher-student proximity increased the cumulative effect of teacher feedback and near-seated peer group influence and increased the buffering effect of the teacher’s positive feedback on the negative effects of disengaged near-seated peer groups. There was asymmetry in the interactive effects between teacher feedback and near-seated peer group influence. The compensating effect of near-seated, high-engagement peer groups on the teacher’s negative feedback is more apparent than that of the teacher’s positive feedback on near-seated, low-engagement peer groups. This might be because the frequency of interactions of students with their peers in this study was higher than that of teacher-student interactions, leading to a stronger influence of near-seated peer groups than teacher feedback. In addition, the positive effect of near-seated peer groups on classroom engagement drove the teacher’s positive feedback to promote classroom engagement, which also partially explains why near-seated peer groups had more significant compensation effects. However, in the low- and high-engagement homogenous and heterogeneous groups, the driving effect in the opposite direction was not found. When near-seated peers had a consistently low or high engagement probability, they might first form stable, close peer relationships due to their similar interactions. Inconsistency in heterogeneous groups might promote the formation of stable, hostile peer relationships. Both friendly and hostile relationships could hinder teacher feedback effects.

Therefore, it is not enough to foster classroom engagement simply by increasing teachers’ positive feedback or boosting the engagement level of near-seated peers: it is important to consider their joint effects. Further, the proximity effect between teachers and students could be utilized to enhance students’ classroom engagement. Teachers could try to predict the emergence of disengaged peer groups in the physical space of the classroom based on the above results and intervene in a timely way.

### Limitations and future research

Our study has several limitations. First, the impact of seating position on classroom engagement included both seat location and self-selection effects [18]. Our study focused on the effect of spatial proximity, and students were randomly assigned in a row-by-column setup. In many cases, students can choose their own seating position. Future research could examine the joint roles of self-selection seating, teacher feedback, and peer group influence and consider other seating arrangements, such as semi-circle arrangements.

Second, we did not explore co-evolutionary mechanisms of the interpersonal relationships and classroom engagement. This study found that the compensative effects of teacher feedback and near-seated peer group influence were asymmetrical, which might be caused by the interactive effect of teacher-student and peer relationships. Teacher support is important for building a good teacher-student relationship, which can help form good peer relationships. When the teacher generally supported more students, the students liked each other more; however, when the teacher supported specific students, they were disliked by their peers [38]. In the future, studies could continue to explore how teacher-student, peer relationships, and classroom engagement coevolve and interact in relation to each other.

Finally, some results need further examination through empirical research. For example, classroom engagement showed an S-shaped growth trend as the teacher’s positive feedback increased, and teacher feedback and near-seated peer group showed asymmetric compensative effects. Ways to effectively balance these two effects to optimize classroom engagement should be further explored.

## Conclusions

Based on previous empirical research, we constructed an agent-based model to examine the influence mechanism of teacher-student proximity, teacher feedback, and near-seated peer groups on classroom engagement. The model simulated the student’s engagement trend close to the observation result and validated the model’s internal mechanism. The study scrutinized the research hypothesis through sensitivity analysis and obtained the following conclusions: (1) As the teacher’s positive feedback increased, classroom engagement increased with an S-shaped curve, and the closer the distance to the teacher, the greater the increase. (2) A higher engagement level of near-seated peer groups contributed to a higher classroom engagement rate, and the difference in the engagement level of near-seated peer groups was positively associated with the difference in classroom engagement rate. (3) The teacher-student proximity enhanced the cumulative effect of teacher feedback and near-seated peer groups on classroom engagement, as well as the compensation effect of teacher feedback. There was asymmetry in the interactions between teacher feedback effect and near-seated peer group influence. The above results imply the joint effect of teacher-student proximity, teacher feedback, and near-seated peer groups on classroom engagement. According to the developmental characteristics of classroom engagement, teachers’ practice could be more effective in improving classroom engagement in classroom teaching and management. They could balance the accumulation and compensation effects of teacher-student proximity, teacher feedback, and near-seated peer groups, and make the interplay of teacher feedback and near-seated peer group influence play a role in promoting students’ engagement and motivational growth.

## Supporting information

S1 Appendix The suitability of ABM. (DOCX)

S2 Appendix Sensitivity analysis of other factors. (DOCX)

S1 Dataset Minimal data set. (ZIP)
