# Supplementary material for: The influence of teacher-student proximity, teacher feedback, and near-seated peer groups on classroom engagement: An agent-based modeling approach
Source: PLoS One. 2021 Jan 7;16(1):e0244935. doi: 10.1371/journal.pone.0244935 (PMC7790242; doi:10.1371/journal.pone.0244935)
Supplement: S1 Appendix — (DOCX) [file pone.0244935.s001.docx]

**S1 Appendix**

**Suitability of ABM**

***What is ABM***

Agent-based modeling (ABM) is a computational methodology that enables one to model complex systems. In agent-based models, individuals or agents are described as unique and autonomous entities that usually interact with other agents or local environment. Agents can be organisms, human beings, enterprises, institutions, and any other entity pursuing specific goals. They have the characteristics of uniqueness, interacting with local and autonomy. Uniqueness means that the agent is usually different from other agents in characteristics such as size, location, resource reserves and history. Interacting with local means that the agent usually does not interact with all other agents, but only with its neighbors in geographic space or other types of "spaces" (such as networks). Autonomy means that the agent act independently of each other and pursue their own goals. Therefore, the agent uses adaptive behavior: the agent adjusts their behavior according to the current state of itself, other agents, and their environment.

***Why use ABM in educational research***

Most previous studies used qualitative, quantitative, or a combination of both methods to examine the impact of seating arrangements on student classroom behavior and learning outcomes [1–3]. However, in educational research, existing quantitative and qualitative methods, either alone or in combination, are not actually sufficient to provide appropriate information and understanding of the dynamics of educational system [4]. The key challenge comes from the non-linearity and emergence of complex systems of education, because macro-level emergence phenomenon is not a simple addition of micro-level parts. Therefore, research needs other technologies suitable for studying complex systems to complement traditional educational research methods.

Maroulis et al. [5] found that visualization and computer simulation can be used as research tools for complex education systems. The visualization of longitudinal network data can link survey or observation data with emerging results. However, these analytical tools are generally used to analyze and explain what has emerged. In this case, ABM is effective because it focuses on micro-level interactions-for which the rules can be obtained through quantitative and qualitative research-and then run the model to explain the various possible results. Furthermore, The combination of ABM with quantitative and qualitative research is thought to have the potential to reveal the dynamic of complex education systems across the range of levels and time scales [4]. The researchers have tried to use ABM to explore educational research questions. For example, [6] utilized ABMs to study initiatives to provide parents with school choice in the United States. More recently, [7] investigated the consequences and efficiency of seating arrangements on academic outcomes and prejudice using an agent-based modeling.

***Appropriateness of ABM for our research***

Based on the characteristics of ABM and the study purpose, we discussed ABM’s potential to model classroom engagement phenomenon from six aspects: Medium numbers, heterogeneous agents, adaptive agents, complex interactions, rich environments, and temporal aspects [8], which determine .

*Medium numbers*: In this case, we are interested to exploring classroom engagement evolution in the classes with 25, 49 and 81 students and some teachers. Although the number is not very large, the modelling results were consistent under the same condition.

*Heterogeneous agents*: The student agents differ in the probability of engagement and seating positions and the teacher agents differ in the probability of positive feedback. The heterogeneity contributed to the changes in the classroom engagement.

*Adaptive agents*: In the model, the students adjust engagement behavior according to previous engagement probability.

*Complex interactions*: The model as proposed has interactions among the teacher, students and near-seated peer groups. In keeping with the reality, students not only interact locally with peer neighbors but also with the teacher in the front.

*Rich environments*: Although the environment in the classroom engagement evolution process does not incorporate all physical and interpersonal environmental factors, seating positions and links among students, teachers and near-seated peers varies between individuals, and thus the environment can be quite rich.

*Temporal aspects*: In this case, we are interested in how classroom engagement evolves under the impacts of teacher-student proximity, teacher feedback and near-seated peer group, so the requirement is met.

Clearly then ABM is an appropriate approach for understanding the classroom engagement development phenomenon at hand. We used NetLogo 6.1.1 developed by Uri Wilensky to build the agent-based classroom engagement model. It was designed to be “low-threshold” — that is, novices can quickly employ it to do meaningful and useful things — but also “high-ceiling” — meaning that scientists and researchers can use it to design cutting-edge scientific models.

**References**

1. Montello DR. Classroom seating location and its effect on course achievement, participation, and attitudes. J Environ Psychol. 1988;8(2): 149–157. http://doi.org/10.1016/S0272-4944(88)80005-7

2. Haghighi MM, Jusan MM. Exploring students behavior on seating arrangements in learning environment: a review. Procedia - Soc Behav Sci. 2012;36: 287–294. http://doi.org/10.1016/j.sbspro.2012.03.032

3. Wannarka R, Ruhl K. Seating arrangements that promote positive academic and behavioural outcomes: A review of empirical research. Support Learn. 2008;23(2): 89–93. http://doi.org/10.1111/j.1467-9604.2008.00375.x

4. Jacobson MJ, Levin JA, Kapur M. Education as a complex system: conceptual and methodological implications. Educ Res. 2019;48(2): 112–119. http://doi.org/10.3102/0013189X19826958

5. Maroulis S, Guimerà R, Petry H, Stringer MJ, Gomez LM, Amaral LAN, et al. Complex systems view of educational policy research. Science (80- ). 2010;330: 38–39. http://doi.org/10.1126/science.1195153

6. Maroulis S, Bakshy E, Gomez L, Wilensky U. Modeling the transition to public school choice. J Artif Soc Soc Simul. 2014;17(2). http://doi.org/10.18564/jasss.2402

7. Radó M, Takács K. Relational integration in schools through seating assignments. Jasss. 2019;22(4). http://doi.org/10.18564/jasss.4115

8. Rand W, Rust RT. Agent-based modeling in marketing: guidelines for rigor. Int J Res Mark. 2011;28(3): 181–193. http://doi.org/10.1016/j.ijresmar.2011.04.002
