# Supplementary material for: The influence of teacher-student proximity, teacher feedback, and near-seated peer groups on classroom engagement: An agent-based modeling approach
Source: PLoS One. 2021 Jan 7;16(1):e0244935. doi: 10.1371/journal.pone.0244935 (PMC7790242; doi:10.1371/journal.pone.0244935)
Supplement: S2 Appendix — (DOCX) [file pone.0244935.s002.docx]

**S2 Appendix**

**Sensitivity analysis of other factors**

### *Influence of Class size*

The class size controlled for the number of students. The front and back rows represented teacher-student proximity. As shown in Table 1 and Fig 1, the size of the class had a small effect on classroom engagement of the entire class. The results of Tamhane T2 test showed that there is no significant difference in classroom engagement rate by class size. However, there was a significant difference between student engagement in the front and back rows, and the difference increased with the increase in class size. In each class, students’ classroom engagement of the front rows was higher than that of the back rows (25: t_29_ = 3.488, p < .01, two-tailed, d = 1.29; 49: t_29_ = 7.201, p < .001, two- tailed, d = 1.84; 81: t_29_ = 14.767, p < .001, two-tailed, d = 5.20).

**Table 1. Means and standard deviations of classroom engagement for class size effect**

|  | Front rows | | Back rows | | The entire class | |
| --- | --- | --- | --- | --- | --- | --- |
| Class size | M | SD | M | SD | M | SD |
| 25 | 79.59 | 7.47 | 72.76 | 7.25 | 75.49 | 5.12 |
| 49 | 80.47 | 4.91 | 69.63 | 5.91 | 74.28 | 3.69 |
| 81 | 83.44 | 3.20 | 68.38 | 4.35 | 75.07 | 2.72 |

**Fig 1. Simulated classroom engagement of front and back rows and the entire class under class size effect.**

### *Effects of teacher-student and peer relatedness thresholds*

The study controlled for the teacher-student and peer relatedness thresholds, of which the range is -10-10. The value of the relatedness thresholds represents the sensitivity of students to the positive and negative influences of teachers' feedback and near-seated peer groups. The higher the threshold, the weaker the sensitivity. The study examined the impact of positive relatedness thresholds on classroom engagement and the negative ones were set to 0. The results showed that classroom engagement rate decreased as the thresholds for teacher-student and peer relatedness increased (Table 2 and Fig 2).

**Table 2. Means and standard deviations of classroom engagement for relatedness threshold effects**

|  | Front rows | | Back rows | | The entire class | |
| --- | --- | --- | --- | --- | --- | --- |
| Parameters | M | SD | M | SD | M | SD |
| Teacher-student relatedness threshold | | | | | | |
| 1 | 82.49 | 4.93 | 72.85 | 5.27 | 76.98 | 3.90 |
| 3 | 81.89 | 5.07 | 71.97 | 5.38 | 76.22 | 3.74 |
| 5 | 80.92 | 5.14 | 69.75 | 4.92 | 74.53 | 3.97 |
| 7 | 77.44 | 4.98 | 66.16 | 4.53 | 70.99 | 3.41 |
| 9 | 70.87 | 4.27 | 57.43 | 5.44 | 63.19 | 3.55 |
| Peer relatedness threshold | | | | | | |
| 1 | 79.68 | 6.54 | 68.61 | 7.53 | 73.35 | 6.13 |
| 3 | 78.64 | 6.35 | 68.73 | 8.10 | 72.97 | 6.53 |
| 5 | 78.93 | 6.74 | 68.26 | 7.27 | 72.83 | 6.08 |
| 7 | 78.73 | 6.44 | 67.50 | 7.39 | 72.31 | 6.21 |
| 9 | 77.63 | 6.35 | 65.05 | 7.06 | 70.44 | 6.00 |


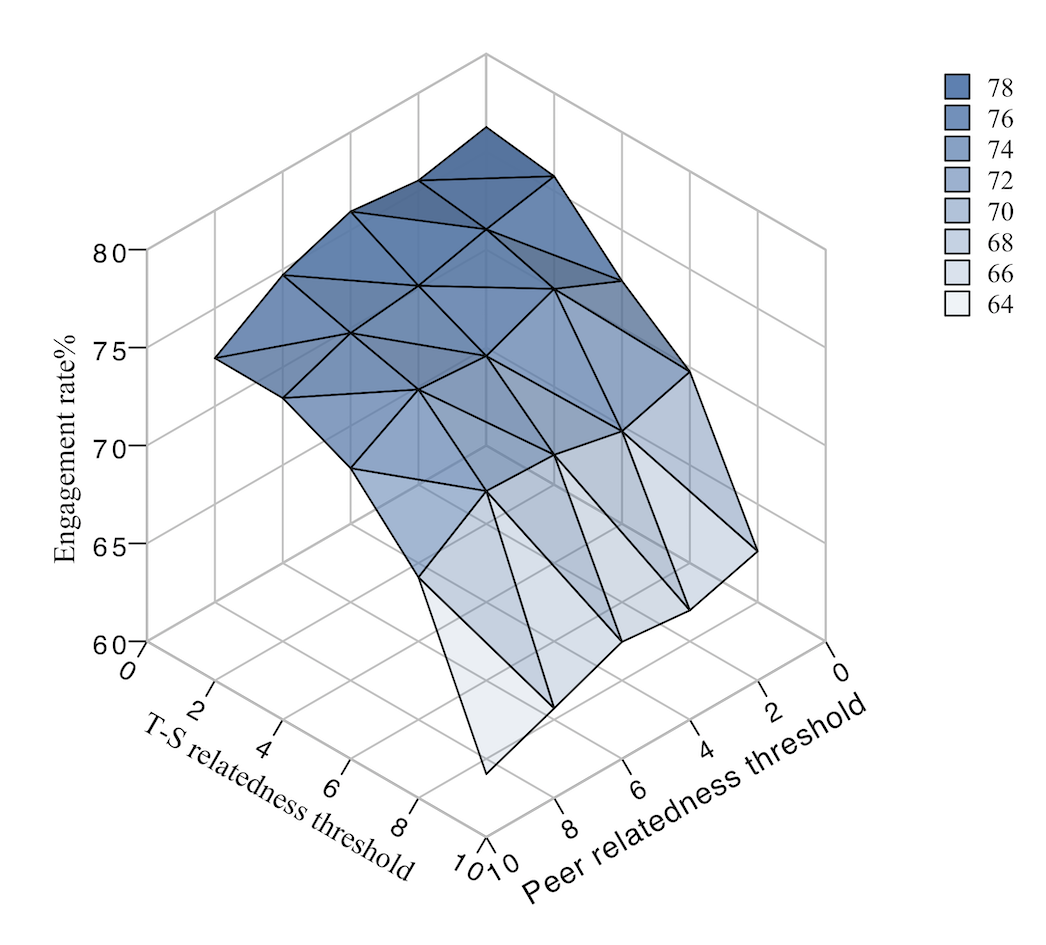

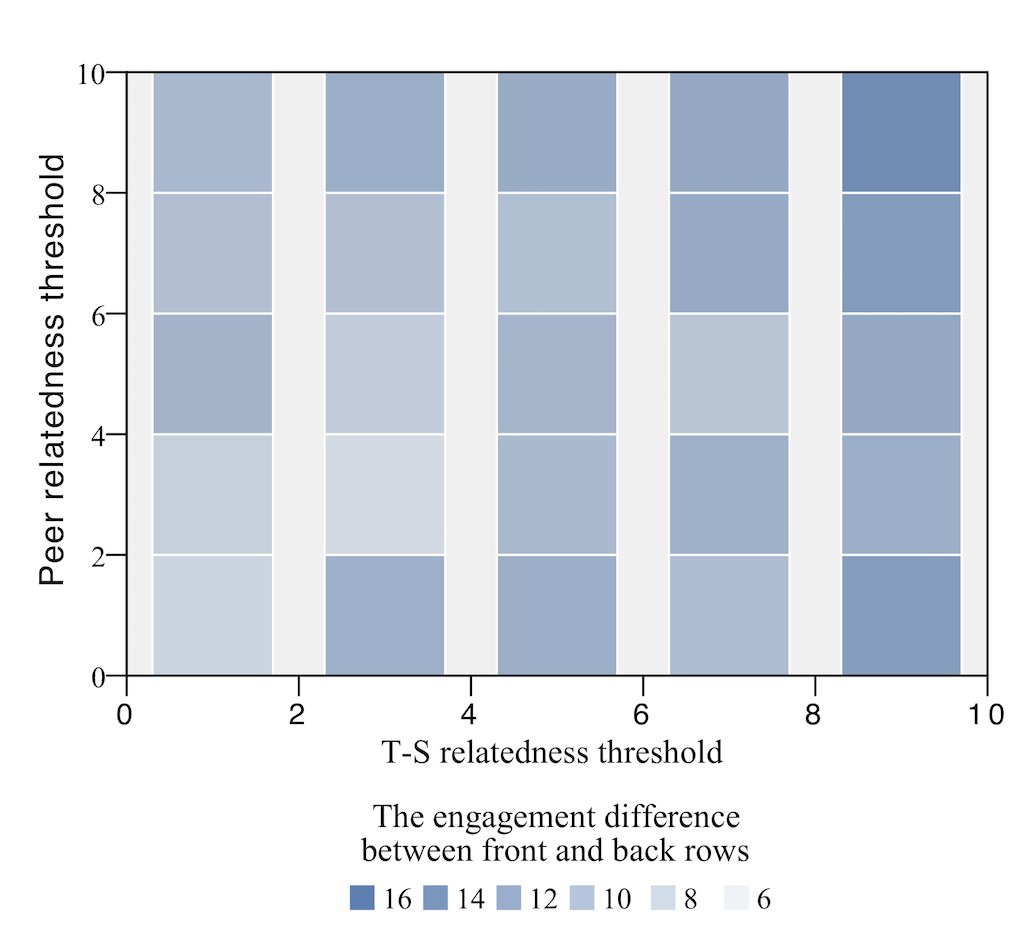


**Fig 2. Simulated classroom engagement outcomes under the influence of teacher-student and peer relatedness thresholds.** Classroom engagement outcomes include (A) classroom engagement of the entire class and (B) the engagement differences between the front and back rows.

The analysis of variance showed that the teacher-student and peer relatedness thresholds have significant effects on classroom engagement of the entire class (F_4, 725_ = 369.273, p < .001, η^2^ = .671；F_4, 725_ = 15.329, p < .001; η^2^ = .078). A post hoc LSD test of threshold differences indicated no significant difference between 1 and 3 levels of teacher-student relatedness threshold (p = .068) and significant differences in other cases (p < .001). Classroom engagement under 1-level peer relatedness threshold was higher than the one under 7-level peer relatedness threshold (p < .05) and classroom engagement for the entire class under 9-level peer relatedness threshold was lower than classroom engagement under other levels (p < .001). Although the interaction between teacher-student and peer relatedness thresholds was not significant (p = .520), when the thresholds were both at level 1, the collective classroom engagement was highest (78.26 ± 3.85), and when both were at level 9, the collective classroom engagement was lowest (63.19 ± 3.55).

The research examined the impact of teacher-student and peer relatedness thresholds on the differences of seating positions under the combination of each level of teacher-student relatedness threshold and each level of peer relatedness threshold. The paired T-test results showed that the differences of student engagement between the front and back rows were significant under all the conditions (p < .001, d ≥ 1.57).

In sum, the effect of class size on classroom engagement was not obvious but the more proximity of students to the teacher (front rows) in each class predicted higher classroom engagement. The relatedness thresholds and teacher-student proximity showed a significant impact on classroom engagement. It showed that lower relatedness thresholds and closer teacher-student proximity contributed to higher classroom engagement.
